# Longitudinal multi-omic dynamics in hospitalized COVID-19 patients based on disease severity

**DOI:** 10.1080/29933935.2026.2712728

**Published:** 2026-08-13

**Authors:** Christopher M. Basting, Ty A. Schroeder, Robin Shields-Cutler, Erik Swanson, Candace Guerrero, Charlotte R. Hemmila, Courtney A. Broedlow, Ashma Chakrawarti, Adrian Velez, Ross Cromarty, Agostino Riva, Alessandro Torre, Alessia Lai, Luca Schifanella, Nichole R. Klatt

**Affiliations:** a Department of Surgery, Division of Surgical Outcomes and Precision Medicine Research, University of Minnesota, Minneapolis, MN, USA; b Department of Biology, Macalester College, St. Paul, MN, USA; c Department of Biochemistry, Molecular Biology and Biophysics, Center for Metabolomics and Proteomics, University of Minnesota, St. Paul, MN, USA; d Masonic Cancer Center, University of Minnesota, Minneapolis, MN, USA; e Department of Infectious Diseases, ASST Fatebenefratelli Sacco, Luigi Sacco Hospital, Milan, Italy; f Department of Biomedical and Clinical Sciences, University of Milan, Milan, Italy; g National Institutes of Health, National Cancer Institute – Center for Cancer Research, Animal Models and Retroviral Vaccines Section, Bethesda, MD, USA

**Keywords:** COVID-19, microbiome, cytokines, fatty acids, bile acids, multiomics, microbial translocation, inflammation

## Abstract

Despite a decline in global COVID-19 cases, severe disease requiring hospitalization remains a significant health burden. Microbial dysbiosis and microbial translocation have been implicated in COVID-19 severity through their contributions to systemic inflammation, yet the temporal dynamics of the microbiome and related metabolites across disease severity are not well defined. To address this, we conducted a longitudinal study of 22 hospitalized COVID-19 patients in Milan, Italy, classified as moderate, severe, or critical by the WHO criteria. Rectal and nasal microbiomes, plasma cytokines, bile acids, fatty acids, and gut barrier damage markers were measured at up to three timepoints over an average of 9 d. Critically ill patients exhibited sustained elevations in pro-inflammatory cytokines (IL-6, IL-8, and TNFα), increased gut barrier damage markers (LBP, zonulin, and sCD14), early depletion of beneficial commensals (including Faecalibacterium prausnitzii), and expansion of opportunistic pathogens such as Hungatella hathewayi and Erysipelatoclostridium ramosum. These microbial shifts were accompanied by the progressive loss of secondary and conjugated bile acids and increased levels of branched- and medium-chain fatty acids. Correlation analyses linked commensal taxa to reduced gut barrier damage and opportunistic pathogens to IL-6. Together, these findings define distinct trajectories associated with COVID-19 severity and highlight the importance of early interventions targeting microbial dysbiosis.

## Introduction

Although the World Health Organization (WHO) declared an end to the COVID-19 pandemic in May 2023,^1^ severe cases leading to hospitalization and death remain a global concern. Tracking the current global health burden of COVID-19 has become more difficult due to changes in reporting practices, but preliminary CDC estimates suggest that between October 2024 and May 2025, the United States (US) experienced approximately 270,000–430,000 hospitalizations and 31,000–50,000 deaths related to COVID-19.^2^ The development of Long COVID is also associated with acute illness severity^3^ and remains a serious public health concern with approximately 7% of the US population having experienced Long COVID symptoms.^4^ Therefore, efforts to understand the factors contributing to severe illness in COVID-19 remain essential for reducing mortality and healthcare burden.

Although most COVID-19 cases are asymptomatic or mild, severe infections are associated with a dysregulated immune response characterized by cytokine storms, lymphopenia, and endothelial dysfunction, which can lead to multi-organ failure and death.^5-8^ Numerous studies have investigated the determinants of COVID-19 severity, highlighting a complex interplay between host, viral, and environmental factors.^5^ ^,^ ^9^ ^,^ ^10^ Older adults and individuals with comorbidities such as obesity, diabetes, or cardiovascular disease are at increased risk, as are male compared to female patients.^11^ ^,^ ^12^ Emerging evidence also implicates disruptions to the nasopharyngeal and gut microbiome in promoting systemic inflammation, which may contribute to more severe disease outcomes.^13-20^

The connection between immunologic biomarkers, COVID-19 severity, and the microbiome is especially compelling to investigate because the gut microbiome is modifiable.^21-23^ While numerous studies have identified associations between microbial compositions and disease severity,^18^ ^,^ ^24^ ^,^ ^25^ most have been cross-sectional, limiting the ability to determine causality and to control for the natural heterogeneity in human microbiome compositions. It remains unclear whether an altered gut microbiome predisposes individuals to severe COVID-19, or if severe illness itself drives dysbiosis, thereby exacerbating the disease through additional inflammation and immune dysregulation.^26^ Likely, both are happening to some degree, as we and others have shown that modulation of the intestinal glycocalyx by the gut microbiome predisposes individuals in an age- and sex-dependent manner to viral infection, as well as linking gut barrier damage and opportunistic pathogens such as *Hungatella hathewayi* to fatal COVID-19 infections and plasma IL-6 concentrations.^17^ ^,^ ^20^ ^,^ ^27^

The microbiome is known to produce and modify a number of metabolites that are critical for host homeostasis, such as short-chain fatty acids (SCFAs), medium-chain fatty acids (MCFAs), and bile acids.^28-30^ COVID-19 has previously been shown to influence the production of some of these metabolites. SCFAs typically decrease with COVID-19 severity while MCFAs, primarily derived from diet, appear to have a more complex relationship where some increase with severity and others do not; overall, these associations with COVID-19 remain understudied and not well understood.^31-33^ Total bile acids in serum are typically increased in severe disease, but a reduction in secondary bile acids in stool is associated with poor outcomes such as respiratory failure.^34^ ^,^ ^35^ Longitudinal studies monitoring immune, virological, microbial, and metabolomic features are essential for fully elucidating the temporal and molecular mechanisms that differentiate patients with critical illness from those with mild disease.

Here, we report a longitudinal observational study of 22 patients hospitalized with COVID-19 classified as having moderate, severe, or critical illness according to WHO guidelines. We then performed comprehensive multi-omic analysis, integrating rectal and nasal microbiomes, viral loads, and plasma levels of bile acids, fatty acids, cytokines, and markers of gut barrier damage, to compare the temporal differences across disease severity groups.

## Results

### Patient characteristics and sampling

Twenty-two patients were enrolled in this study, with up to three samples collected over the course of hospitalization. There were no significant differences between the groups in how many days passed between when samples were collected (ANOVA *p* > 0.05, Supplementary Figure 1B and 1C). Most of the patients enrolled were men (77.3%), and although there were no significant differences in the proportions of men versus women between severity groups (Fisher’s exact *p* = 0.3807, Supplementary Figure 1D), both the moderate and severe groups included some women, whereas the critical group included only men. The available samples collected at each time point for each patient are shown in Supplemental Table 1, with the serological and viral load summary statistics for each group shown in Supplemental Table 2. As no healthy participants were included in this study, normative values for plasma cytokines, fatty acids, and bile acids can be referred to from previous literature.^36-38^ In general, inflammatory cytokines (e.g., IL-6, IL-8, and TNFα) were increased compared to normative values.

### Gut barrier damage and microbial translocation biomarkers remain elevated in critical COVID-19 patients

**Figure 1.**
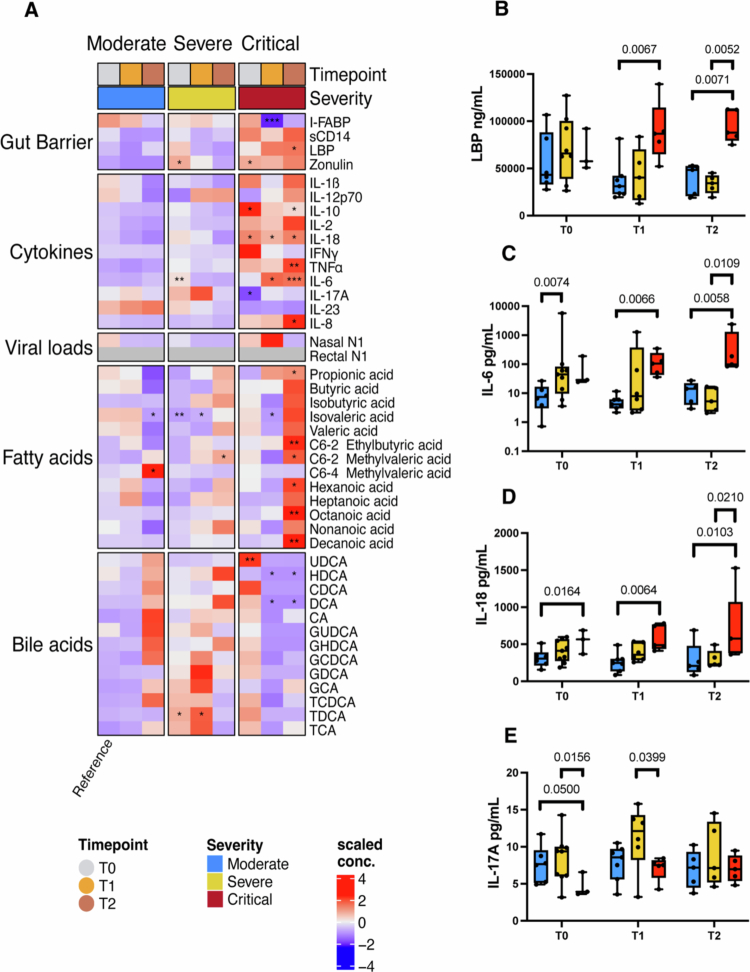
Longitudinal differences in the plasma concentrations of gut barrier integrity markers, cytokines, fatty acids, bile acids, and nasal/rectal swab SARS-CoV-2 viral loads between hospitalized COVID-19 patients with varying severity. Heatmap of the scaled median concentration of each variable by severity and timepoint (A). Rectal swab N1 viral loads are NA because all the median concentrations are equal to zero prior to scaling. Asterisks represent the *p*-value associated with each effect from the linear mixed effects model with moderate: T0 as the reference level: *p < 0.05, **p < 0.01, ***p < 0.001. Representative box plots for LBP (B), IL-6 (C), IL-18 (D), and IL-17A (E). Box plot whiskers extend to the min and max values, with the median plotted in the center of the box.

Longitudinal differences in biomarker concentrations between degrees of COVID-19 severity were compared using linear mixed effects models (Figure 1A, Supplemental Table 3), with pairwise comparisons at each time point (Supplemental Table 4). For biomarkers of microbial translocation, the levels of lipopolysaccharide-binding protein (LBP) were significantly higher in critically ill patients compared to moderately ill patients at both T1 and T2 (*p* = 0.0067 and *p* = 0.0071) and compared to severely ill patients at T2 (*p* = 0.0052; Figure 1B). Similarly, soluble CD14 (sCD14) remained comparable across groups until T2, when they were significantly elevated in critically ill patients relative to both moderate and severe cases (*p* = 0.0275 and *p* = 0.0348; Supplemental Figure 2A). For biomarkers of gut barrier integrity, intestinal fatty acid binding protein (I-FABP) demonstrated a transient decrease in critically ill patients at T1, with significantly lower concentrations compared to moderate and severe groups (*p* = 0.0041 and *p* = 0.0121; Supplemental Figure 2B), but it returned to the same level at T2. Zonulin levels were elevated in critically ill patients at T0 compared to both the moderate and severe groups (*p* = 0.0431 and *p* = 0.0257) and remained significantly higher at T2 compared to moderately ill patients (*p* = 0.0273; Supplemental Figure 2C). Overall, markers of microbial translocation and gut barrier disruption were elevated in critically ill patients, often earlier in the hospitalization course than in moderate and severe patients, and tended to remain elevated overtime.

### Pro-inflammatory cytokines increase overtime in critical COVID-19 patients

The next class of biomarkers we examined was circulating plasma cytokines. Among these cytokines, the pro-inflammatory cytokine IL-6 showed the most pronounced differences in temporal trajectory across the severity groups. IL-6 levels were significantly elevated in severely ill patients compared to moderately ill patients at T0 (*p* = 0.0074) but declined overtime and were no longer significantly different at T1 or T2. In contrast, IL-6 levels in critically ill patients increased overtime and remained elevated, being significantly higher than those in moderately ill patients at both T1 and T2 (*p* = 0.0067 and *p* = 0.0071) and higher than those in severely ill patients at T2 (*p* = 0.0052; Figure 1C).

Other cytokines exhibited similar patterns, with levels generally declining overtime in moderate and severe cases, while remaining elevated or increasing in critical cases. IL-18 was consistently elevated in critically ill patients and was significantly higher than in moderately ill patients at all time points (*p* = 0.0164, 0.0064, and 0.0103 for T0, T1, and T2, respectively), and higher than in severely ill patients at T2 (*p* = 0.0210; Figure 1D).

At T2, IL-8 and TNFα were significantly elevated in critically ill patients compared to both moderate (*p* = 0.0088 and *p* = 0.0080) and severe cases (*p* = 0.0219 and *p* = 0.0413; Supplemental Figures 2D and 2E, respectively). Interestingly, the regulatory cytokine IL-10 was significantly higher in critically ill patients compared to moderately ill patients at T0 and T2 (*p* = 0.0163 and *p* = 0.0145; Supplemental Figure 2F), potentially reflecting a regulatory response to increased inflammation. The immunomodulatory cytokine IL-2 was also elevated in critical patients at T2 compared to moderate patients (*p* = 0.0423; Supplemental Figure 2G).

Notably, the level of IL-17A, a homeostatic cytokine associated with mucosal immunity, was the only cytokine that decreased in critically ill patients relative to other groups. Levels were significantly lower in critical patients at T0 compared to both moderate and severe patients (*p* = 0.0500 and *p* = 0.0156, respectively). By T1 and T2, critical patient levels had recovered to those of the moderate group, with the continued significant difference at T1 driven by an increase in the severe group (*p* = 0.0399; Figure 1E). IL-23 levels were also lowest in critically ill patients across all time points and highest in moderate patients, although no pairwise comparisons reached statistical significance.

### Fatty acids, especially branched-chain and medium-chain fatty acids, increase in critical COVID-19 patients

**Figure 2.**
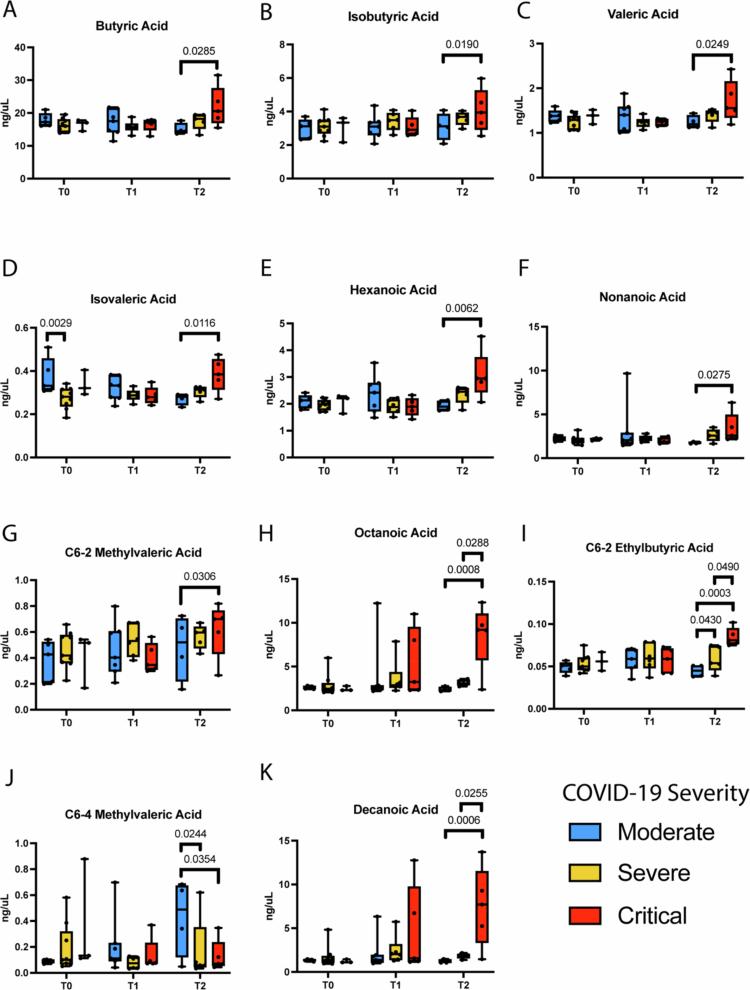
Longitudinal differences in the plasma concentrations of fatty acids between hospitalized COVID-19 patients with varying severity. Concentrations of butyric acid (A), isobutyric acid (B), valeric acid (C), isovaleric acid (D), hexanoic acid (E), nonanoic acid (F), C6-2 methylvaleric acid (G), octanoic acid (H), C6-2 ethylbutyric acid (I), C6-4 methylvaleric acid (J), and decanoic acid (K). Pairwise comparisons between groups at each time point were performed using the emmeans R package, and nominal *p*-values < 0.05 were considered significant. Box plot whiskers extend to the min and max values with the median plotted in the center of the box.

We next evaluated circulating concentrations of short-chain and medium-chain fatty acids, including several branched-chain derivatives. The most notable differences were observed between moderately and critically ill patients at T2. Several fatty acids were significantly elevated in critically ill patients at T2 compared to moderately ill individuals, including butyric acid (*p* = 0.0285; Figure 2A), isobutyric acid (*p* = 0.0190; Figure 2B), valeric acid (*p* = 0.0249; Figure 2C), isovaleric acid (*p* = 0.0116; Figure 2D), hexanoic acid (*p* = 0.0062; Figure 2E), nonanoic acid (*p* = 0.0275; Figure 2F), and 2-methylvaleric acid (*p* = 0.0306; Figure 2G).

Octanoic acid levels were elevated in critically ill patients compared to both moderate and severe groups at T2 (*p* = 0.0008 and *p* = 0.0288; Figure 2H). Furthermore, 2-ethylbutyric acid was increased in both severe and critically ill patients compared to moderate cases at T2 (*p* = 0.0430 and *p* = 0.0003), and its levels were also significantly higher in critical versus severe cases (*p* = 0.0490; Figure 2I). In contrast, 4-methylvaleric acid was decreased in severe and critical patients relative to moderate patients at T2 (*p* = 0.0244 and *p* = 0.0354; Figure 2J). Finally, decanoic acid was significantly elevated in critically ill patients compared to both the moderate and severe groups at T2 (*p* = 0.0006 and *p* = 0.0255; Figure 2K). We next examined total SCFA, BCFA, and MCFA concentrations overtime (Supplemental Figure 3), finding that all three classes were elevated in critical patients compared to moderate patients at T2, with MCFAs additionally elevated compared to severe patients (*p* < 0.05). Overall, the most striking pattern we observed was an increase in circulating fatty acids in critically ill patients at the final time point, especially for branched- and medium-chain fatty acids.

### Loss of bile acid pool in critically ill COVID-19 patients

**Figure 3.**
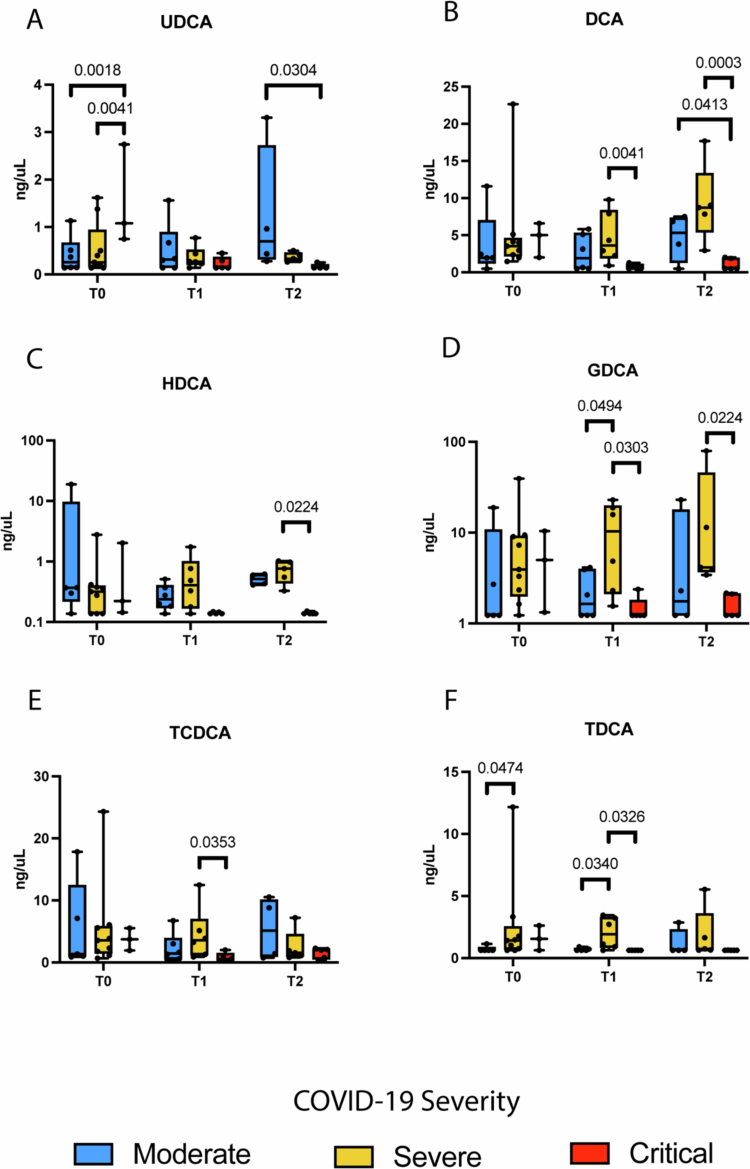
Longitudinal differences in the plasma concentrations of bile acids between hospitalized COVID-19 patients with varying severity. Concentrations of UDCA (A), DCA (B), HDCA (C), GDCA (D), TCDCA (E), and TDCA (F). Pairwise comparisons between groups at each time point were performed using the emmeans R package, and nominal *p*-values < 0.05 were considered significant. Box plot whiskers extend to the min and max values, with the median plotted in the center of the box. Ursodeoxycholic acid (UDCA), deoxycholic acid (DCA), hyodeoxycholic acid (HDCA), glycodeoxycholic acid (GDCA), taurochenodeoxycholic acid (TCDCA), and taurodeoxycholic acid (TDCA).

We next measured the circulating concentrations of several primary, secondary, and conjugated bile acids. The most pronounced differences were observed for the secondary bile acids ursodeoxycholic acid (UDCA) and deoxycholic acid (DCA). UDCA levels were significantly elevated in critically ill patients at T0 compared to both moderately and severely ill patients (*p* = 0.0018 and *p* = 0.0041), which was reversed by T2, with moderate patients having significantly elevated UDCA compared to critical patients (*p* = 0.0304; Figure 3A). In contrast, DCA levels were higher in moderately and severely ill patients relative to critically ill patients, with significant differences between severe and critical patients at T1 (*p* = 0.0041) and between critical and both moderate and severe groups at T2 (*p* = 0.0413 and *p* = 0.0003; Figure 3B).

Additional differences were observed among conjugated bile acids. Hyodeoxycholic acid (HDCA) was significantly reduced in critically ill patients at T2 compared to severe cases (*p* = 0.0224; Figure 3C). Glycodeoxycholic acid (GDCA) was elevated in severely ill patients at T1, with significant pairwise differences compared to both moderate and critically ill groups (*p* = 0.0494 and *p* = 0.0303) and remained significantly higher than that in critically ill patients at T2 (*p* = 0.0224; Figure 3D). Taurochenodeoxycholic acid (TCDCA) was also significantly elevated in severely ill patients relative to critically ill patients at T1 (*p* = 0.0353; Figure 3E). Finally, taurodeoxycholic acid (TDCA) was elevated in severely ill patients, with significant differences observed compared to moderately ill patients at T0 (*p* = 0.0474) and in both the moderate and critical groups at T2 (*p* = 0.0340 and *p* = 0.0326; Figure 3F).

### Viral loads are higher in nasal swabs than rectal swabs, but did not differ between COVID-19 severity

To determine whether viral load was associated with disease severity, we quantified SARS-CoV-2 N1 gene copies/μL in both nasal and rectal swabs. We did not observe any significant pairwise differences in viral load across severity groups in either swab type (Supplemental Figures 4A and 4B). Rectal swab viral loads were generally low, with only 11 out of 58 swabs showing detectable N1 gene copy numbers. In contrast, nasal swab viral loads were substantially higher than rectal swab values at both T0 and T1 (*p* < 0.0001 and *p* = 0.0016, respectively) and decreased overtime, with significant differences between T0 and both T1 and T2 time points (*p* = 0.0137 and *p* = 0.0097; Supplemental Figure 4C).

### Temporal dynamics of the rectal and nasal microbiomes differ based on COVID-19 severity

**Figure 4.**
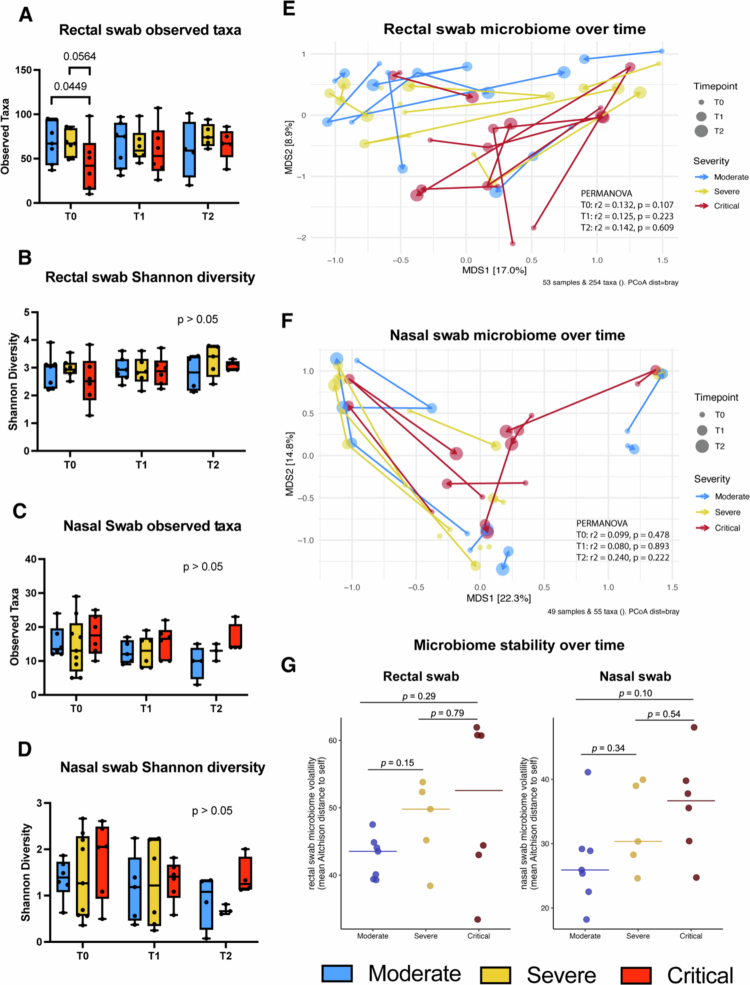
Rectal and nasal microbiome changes overtime across COVID-19 severity groups. Rectal and nasal swab alpha diversity measures overtime for the observed taxa (A, C) and Shannon diversity (B, D). Box plot whiskers extend to the min and max values, with the median plotted in the center of the box. Of note, only 21/22 participants had swabs available for analysis. PCoA of Bray‒Curtis distances for rectal swabs (E) and nasal swabs (F) overtime, where individual patient samples are connected chronologically by lines and timepoints are indicated by circle diameters. PERMANOVA tests were performed individually for each time point. Microbiome stability for rectal and nasal swabs (G) was measured by the mean within-individual Aitchison distance for each sample and tested for significance using pairwise Wilcoxon rank-sum tests.

We then measured and compared the nasal and rectal microbial compositions longitudinally within and between patients. Critical patients had reduced rectal microbial richness at T0 compared to moderate patients (*p* = 0.0449; Figure 4A) and near significance compared to severe patients (*p* = 0.0564). We did not observe any other differences in alpha diversity for other pairwise comparisons, or for the Shannon alpha diversity metric (*p* > 0.05; Figure 4B). We also did not observe any differences in nasal swab alpha diversity between any of the severity groups (*p* > 0.05; Figure 4C and D).

Similarly, there was no significant difference between the severity groups in beta diversity (Bray‒Curtis and Jaccard) for both rectal and nasal swabs as calculated by PERMANOVA at T0, T1, or T2 (*p* > 0.05; Figure 4E and F). Although statistically non-significant, there was a trend toward greater rectal and nasal microbiome composition volatility that increased with disease severity, as measured by the mean within-individual Aitchison distance between timepoints (Figure 4G).

**Figure 5.**
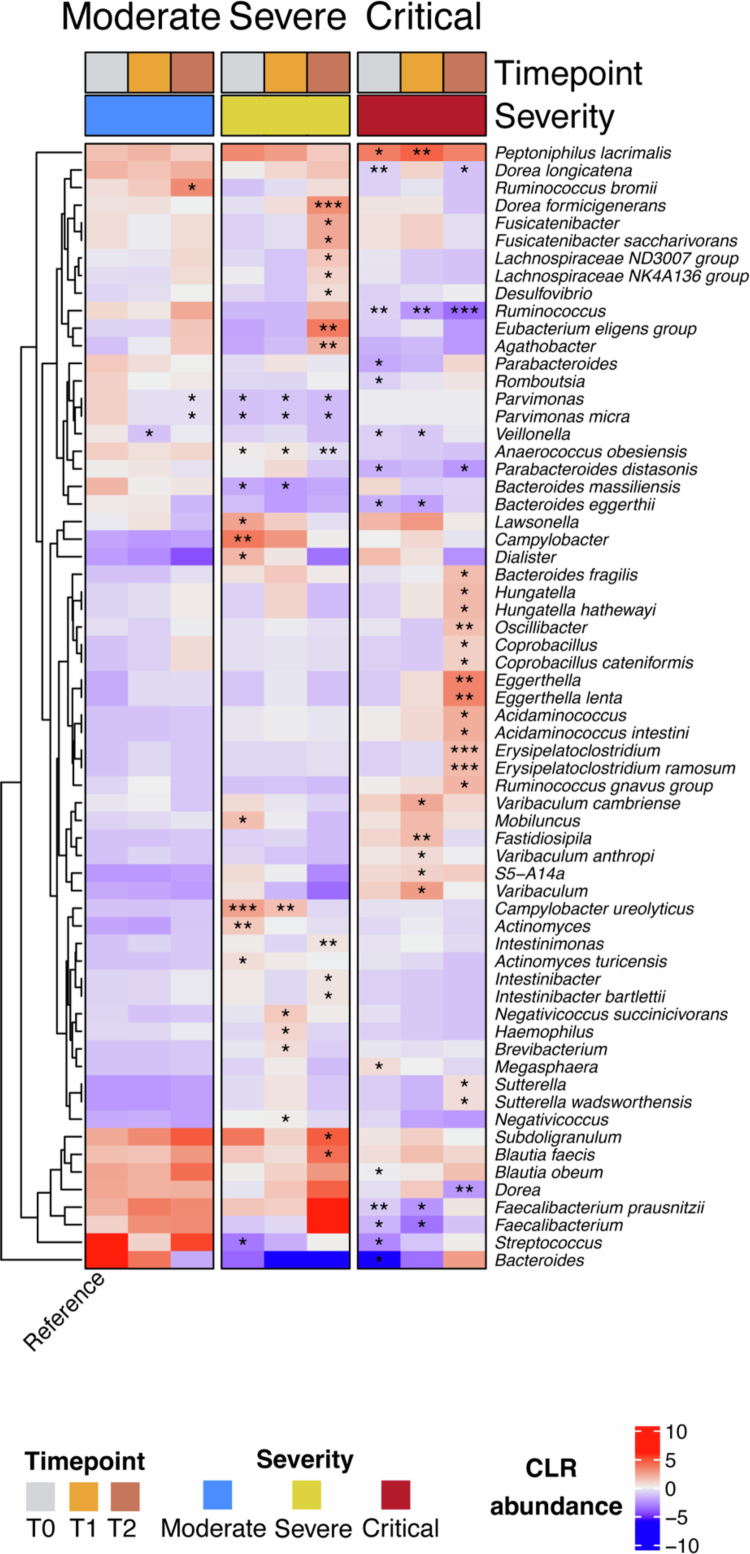
Differential abundance analysis of the rectal swab microbiome. Significant rectal swab taxa from MaAsLin2 analysis (A) using moderate: T0 as the reference level: *p < 0.05, **p < 0.01, ***p < 0.001. Fixed effects for each model included a composite variable of severity-timepoint and gender, with patient ID as a random effect to account for repeated measures.

**Figure 6.**
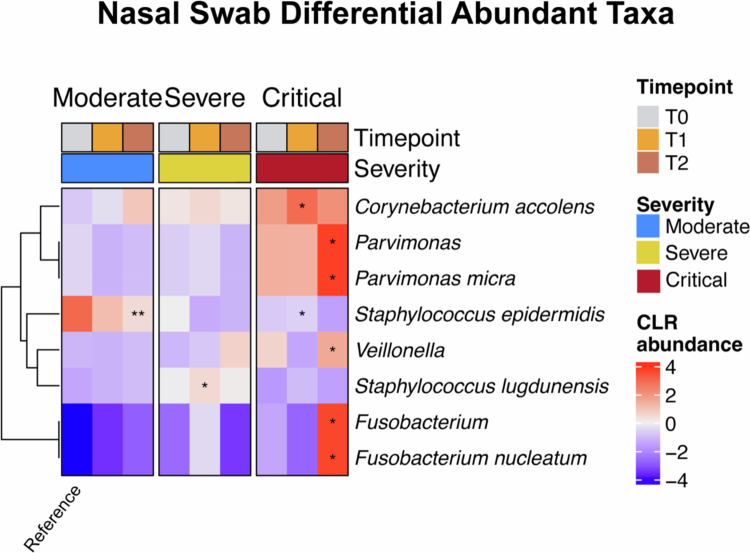
Differential abundance analysis of the nasal swab microbiome. Significant rectal swab taxa from MaAsLin2 analysis (A) using moderate: T0 as the reference level: *p < 0.05, **p < 0.01. Fixed effects for each model included a composite variable of severity-timepoint and gender, with patient ID as a random effect to account for repeated measures.

Next, we performed differential abundance analysis using MaAsLin2 to investigate how the relative abundance of taxa changed overtime in the COVID-19 severity groups, using moderate patients at T0 as the reference for all comparisons. Most notably, critically ill patients had a lower abundance of numerous commensal bacteria at the start of their hospitalization. For example, *the abundances of Ruminococcus, Veillonella, Faecalibacterium prausnitzii, Blautia obeum, Bacteroides eggerthii,* and *Dorea longicatena* were lower at T0 compared to moderate patients (*p* < 0.05; Figure 5). Many of these taxa remained significantly depleted through the hospitalization, or even decreased further, such as in the case of *Ruminococcus*. In contrast, taxa including *Hungatella hathewayi*, *Eggerthella lenta*, *Erysipelatoclostridum ramosum*, *Bacteroides fragilis*, *Coprobacillus cateniformis*, *Acidaminococcus intestine*, *Ruminococcus gnavus group*, and *Sutterella wadsworthensis* were exclusively increased in abundance for the critically ill patients at T2 (*p* < 0.05; Figure 5), many of which are known opportunistic pathogens or have previously associated with COVID-19.^17^ In severe patients we saw the opposite pattern, where commensal short chain fatty-acid producers such as *Dorea formicigenerans*, *Fusicatenibacter saccharivorans*, *Lachnospiraceae ND3007/NK4A136 groups, Subdoligranulum*, and *Blautia faecis* increased overtime and pathogens including *Campylobacter ureolyticus* decreased. Notably, increased *Campylobacter ureolyticus* in severe patients at T0 was the only association that remained significant after multiple comparison adjustment (FDR *p* < 0.05). Moderate patients generally had the fewest changes in taxa overtime. Differentially abundant taxa in the nasal microbiome were less pronounced than those observed in the rectal swabs. Most notably, critical patients had an increased abundance of *Fusobacterium nucleatum*, *Parvimonas micra*, and *Veillonella* at T2 compared to moderate T0 (*p* < 0.05; Figure 6).

### Repeated measures correlation analysis highlights relationships between inflammation, gut barrier damage, and microbial dysbiosis

**Figure 7.**
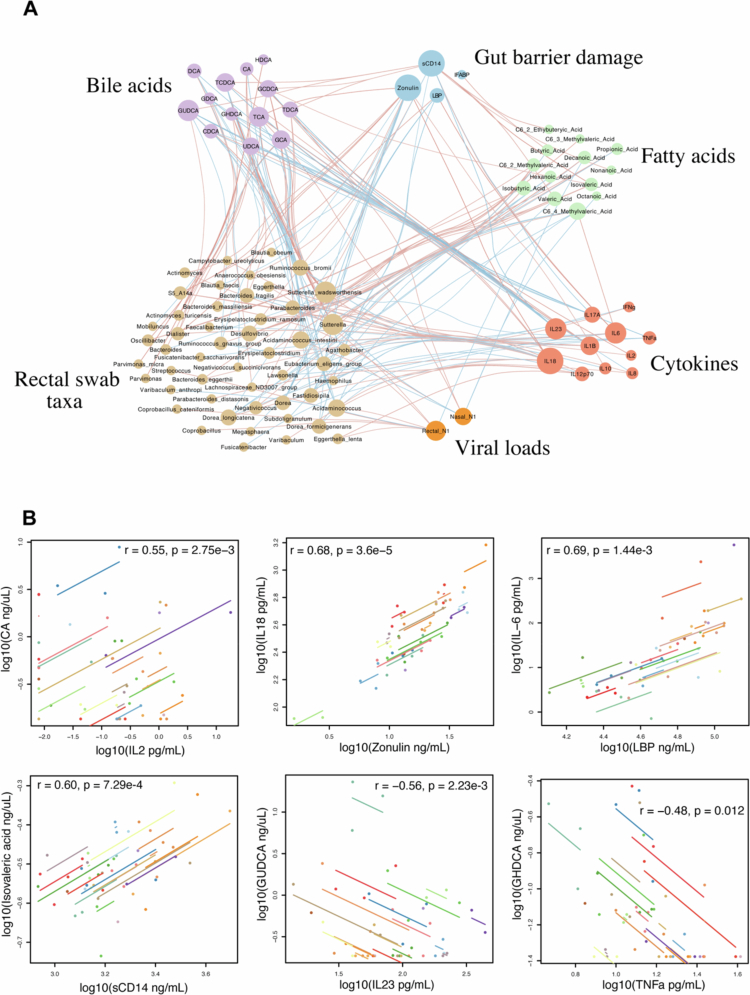
Multi-omic repeated measures correlations analysis of hospitalized COVID-19 patients. Correlations between data types were performed using the *rmcorr* R package, which accounts for repeated samplings overtime. Network showing all significant correlations (nominal *p* < 0.05) with a correlation coefficient greater than 0.4 or less than −0.4 (A). Each edge represents a significant correlation between two variables, with red edges being positive correlations and blue being negative. The size of each node/variable represents its degree in the network, with larger nodes having more edges. Representative scatterplots for correlations of interest (B), where colors denote individual subjects and lines represent subject-specific fitted values assuming a fixed slope and random intercept.

Repeated measures correlations were performed to evaluate the relationships between the rectal microbiome, plasma cytokines, microbial translocation biomarkers, gut barrier integrity biomarkers, and metabolites over time (Figure 7A and B; Supplemental Table 5). Interestingly, we observed negative correlations between several commensal bacteria and both pro-inflammatory cytokines and markers of gut barrier damage. For example, the *Dorea* genus was negatively correlated with plasma IL-6 (*r* = −0.46, *p* = 0.0139), TNFα (*r* = −0.48, *p* = 0.0095), and zonulin (*r* = −0.46, *p* = 0.0132). Similarly, *Ruminococcus bromii* and *Blautia obeum* were negatively correlated with zonulin (*r* = −0.44, *p* = 0.0196 and *r* = −0.44, *p* = 0.0183, respectively). Conversely, several opportunistic pathogens were positively correlated with indicators of disease severity. *Erysipelatoclostridium ramosum* was positively correlated with IL-6 (*r* = 0.40, *p* = 0.0339), IL-8 (*r* = 0.38, *p* = 0.0467), and IL-10 (*r* = 0.37, *p* = 0.0492), while *Eggerthella lenta* abundance was additionally correlated with rectal swab N1 copies/µL (*r* = 0.41, *p* = 0.0181) and IL-12p70 (*r* = 0.49, *p* = 0.0087).

We also observed positive correlations between several markers of gut barrier damage and pro-inflammatory cytokines. Both LBP and Zonulin were positively correlated with IL-18 (r = 0.37, *p* = 0.048 and r = 0.68, *p* = 3.6e^−5^, respectively), and LBP was further positively correlated with IL-6 (r = 0.56, *p* = 0.0014). sCD14 was also negatively correlated with both IL-23 (r = −0.58, *p* = 0.0008) and IL-17 (r = −0.53, *p* = 0.0027). For bile acids, a general trend was that many were negatively correlated with IL-23 and IL-17. UDCA, glycoursodeoxycholic acid (GUDCA), TDCA, and GDCA were all negatively correlated with IL-17A, while GUDCA, TCDCA, TDCA, glycochenodeoxycholic acid (GCDCA), GDCA, DCA, and chenodeoxycholic acid (CDCA) were negatively correlated with IL-23 (*p* < 0.05). Further, *Sutterella wadsworthensis* abundance was positively correlated with many conjugated bile acids, including TCDCA, glycocholic acid (GCA), GCDCA, taurocholic acid (TCA), glycohyodeoxycholic acid (GHDCA), TDCA, and GUDCA (*p* < 0.05). Collectively, these correlations highlight the relationships between the gut microbiome, intestinal barrier damage, mucosal immunity, and systemic inflammation during acute COVID-19 infection.

## Discussion

The longitudinal dynamics of biomarkers in COVID-19 patients can help inform mechanisms driving disease progression and be used to predict patient outcomes and develop therapeutic interventions. Our longitudinal study characterized serological biomarkers (cytokines, microbial translocation markers, gut barrier integrity markers, fatty acids, and bile acids), SARS-CoV-2 viral loads, and the microbiomes of nasal and rectal swabs in 22 patients hospitalized with various degrees of COVID-19 severity. Our analysis reveals distinct temporal patterns of biomarker dynamics across severity groups and highlights key relationships among these biomarkers.

Markers of gut barrier damage were elevated in patients with severe and critical illness, which is consistent with previous findings and reinforcing data from us and others that intestinal barrier integrity is compromised in COVID-19.^17^ ^,^ ^20^ Interestingly, the persistence, or even progression, of these markers over time in critically ill patients suggests a failure to restore gut homeostasis during illness. Zonulin, for example, was elevated early in both the severe and critical groups, but remained high only in the critically ill group at later time points, suggesting sustained barrier dysfunction unique to this group. Similar patterns for LBP and sCD14 further support the notion that microbial translocation may be ongoing in the most ill patients and is contributing to systemic inflammation, as indicated by the positive correlation we observed between plasma LBP and IL-6. These findings highlight the potential value of using these biomarkers to monitor gut barrier health longitudinally in hospitalized populations, an area that remains underexplored in the current literature.

Inflammatory cytokines, particularly IL-6, TNFα, IL-18, and IL-8, were significantly elevated in critically ill patients, primarily at the T2 timepoint, suggesting a progressive increase in inflammatory signaling as disease severity worsens. Of interest, IL-10 and IL-18 were both elevated in critical illness patients at T0 compared to moderate and severe patients, indicating their potential as early markers of disease severity. Notably, the regulation of IL-18 is essential for maintaining gut barrier integrity, with increased concentrations associated with reduced goblet cell maturation, mucus production, and the pathological breakdown of the gut barrier in ulcerative colitis.^39-41^ Its early association with critical COVID-19, along with markers such as zonulin, and the strong positive correlation between IL-18 and zonulin, suggests that disruption of gut mucosal homeostasis may occur early in infection and potentially precede systemic inflammation.

Aside from direct impacts on the upregulation of inflammatory markers, COVID-19 infection is also known to alter host fatty acid profiles,^31^ particularly those derived from microbial metabolism in the gut. In this cohort, critically ill patients had significantly elevated concentrations of branched-chain fatty acids (BCFAs), MCFAs, and modest increases in SCFAs at the T2 time point, relative to those with moderate or severe illness. This finding partially differs from previous reports of reduced SCFAs in COVID-19 patients attributed to dietary shifts and loss of fiber-consuming commensal microbes.^31^ ^,^ ^42^ Although we observed depletion of numerous SCFA-producing bacteria in critical patients, butyric acid and valeric acid concentrations increased over time in these individuals, along with their branched-chain counterparts including isobutyric and isovaleric acid. Interestingly, these fatty acids can be produced via microbial fermentation of branched-chain amino acids, including valine and leucine, especially by *Clostridium* and *Bacteroides* species in high-protein, low-fibre environments.^43^ ^,^ ^44^ *Erysipelatoclostridium ramosum* (formerly *Clostridium ramosum*) was elevated in critical patients at T2 and positively correlated with plasma concentrations of isovaleric acid; however, it has not been reported to produce BCFAs. Given that dietary interventions are common in critical care settings such as the intensive care unit (ICU), and that high-protein diets are typically used to prevent muscle protein catabolism,^45^ one possibility is that the observed shifts in fatty acid profiles may reflect a dietary-driven metabolic shift of the gut microbiome from fiber-based to amino acid-based fermentation, though this is speculative in the absence of dietary intake data. Relatively little is known about BCFAs compared to SCFAs, but studies indicate that fecal concentrations of BCFAs negatively correlate with fiber intake^44^ and that prolonged exposure to BCFAs and other products of amino acid fermentation, such as ammonia and *p*-cresol, negatively affects the viability of colonic epithelial cells.^46^

In contrast to fatty acid profiles, bile acid concentrations decreased over time in critically ill patients, whereas moderate and severe patients maintained or showed slight increases. These differences were most pronounced for secondary and conjugated bile acids, and we did not observe any differences between the primary bile acids cholic acid (CA) and CDCA. Among the most depleted secondary bile acids in critical patients at T2 were DCA, UDCA, and HDCA. Increased concentrations of secondary bile acids in COVID-19 patients in the ICU have previously been reported to correlate with improved survival,^47^ which agrees with our observation that these bile acids are generally elevated in moderate and severe patients compared with critical patients. The farnesoid X receptor (FXR) and Takeda G protein-coupled receptor 5 (TGR5) are primary receptors for bile acids, both of which regulate ACE2 expression and have immunoregulatory functions, often attenuating inflammation.^47^ For this reason, bile acid supplementation, particularly with UDCA, has been explored as a treatment for COVID-19, with mixed results. While some studies suggest that UDCA reduces SARS-CoV-2 susceptibility and COVID-19 severity, others report no benefit on mortality and overall survival.^47^ Our findings indicate that bile acid depletion occurs progressively during hospitalization in the most critically ill patients. Given that secondary and conjugated bile acids are largely produced by the gut microbiota, their depletion possibly reflects the microbial changes we observed, potentially mediated by dietary changes.

The microbial dysbiosis observed in COVID-19 patients here was dependent on disease severity, which is consistent with the findings of previous studies,^24^ ^,^ ^48^ ^,^ ^49^ with higher alpha diversity in the gut microbiome typically associated with better health outcomes.^50-53^ Here, critically ill patients presented significantly lower microbial richness at T0 in the rectal microbiome, which returned to the same level as moderate and severe at T1 and T2. This difference at T0 was also associated with the loss of several important commensal bacteria, including *Faecalibacterium prausnitzii*, *Ruminococcus*, *Dorea formicigenerans*, and *Dorea longicatena,* as well as increased zonulin concentrations. These differences were unique to critically ill patients and were preceded by the predominance of opportunistic pathogens at T2, including *Hungatella hathewayii*, *Eggerthella lenta*, and *Erysipelatoclostridium ramosum*, which have previously been implicated in acute COVID-19.^17^ ^,^ ^54^ ^,^ ^55^ We also observed an increase in *Bacteroides fragilis* in critically ill patients at T2, which has previously been implicated as a predictor of both disease severity and enteral feeding.^56^ These trends are drastically different from what was observed in severely ill patients, who had certain commensals, such as *Subdoligranulum* and *Blautia faecis* increase over time while pathogenic bacteria, such as *Campylobacter ureolyticus*, have decreased. Supporting the role of commensal gut bacteria in gut barrier integrity and systemic inflammation, we observed that *Dorea* was negatively correlated with markers of microbial translocation and pro-inflammatory cytokines. Conversely, the opportunistic pathogen *Erysipelatoclostridium ramosum* was positively correlated with the pro-inflammatory cytokines IL-6 and IL-8. Interestingly, *Sutterella wadsworthensis*, a bile resistant microbe, was positively correlated with seven conjugated bile acids, possibly reflecting this bacterium’s fitness advantage in the presence of bile compared to non-resistant microbes.

The nasal swab microbiome presented fewer differences between severity groups than rectal swabs. Most notably, *Fusobacterium nucleatum* and *Parvimonas micra* were both increased at T2 in critically ill patients. Both of these taxa are well-documented opportunistic pathogens in the oral cavity that have consistently been associated with colorectal cancer.^57-59^ In addition, *Veillonella* is also enriched in nasal swabs of critical patients, and its enrichment is associated with disease states where it can form biofilms with other bacteria, perpetuating dysbiosis and contributing to systemic inflammation.^60^ ^,^ ^61^ Given that the nasal microbiome is unlikely to be altered by dietary interventions, these data support that COVID-19 itself may drive dysbiosis.

The results of this study must be interpreted in the context of its limitations. First, the lack of *p*-value adjustment for multiple comparisons was chosen due to the study's small sample size, high dimensionality, and exploratory nature, which focused on hypothesis generation. Since we report nominal *p*-values across a large number of statistical tests, the risk of Type I error is elevated. An additional limitation is the lack of adjustment for other confounding variables that were unavailable for our analysis due to the nature of early COVID-19 disease overwhelming hospital systems and the urgent nature of sample collection in this study. This includes patient age, diet, comorbidities, interventions during hospitalization, and the exact timing of symptom onset and admittance to the hospital. All these factors could influence the serological biomarkers, microbiome, and viral load variables we measured. We also lacked a pre-infection baseline sample for each patient, so we cannot discern whether changes in the microbiome were in response to infection or possibly predisposed individuals to a specific outcome. Further, the samples used were from cases in which the SARS-CoV-2 alpha variant was dominant and may not align with infections from current viral strains. Of note, given that these samples were collected at the peak of the COVID-19 outbreak in Italy, more granular severity metadata was also unavailable, including survival metrics, and longitudinal trajectories of recovery, which would be an important direction for future studies to consider. Future studies incorporating continuous severity scores may also better capture graded variation in disease severity and improve the identification of global molecular signatures. In addition, acetate was excluded from the SCFA panel due to technical limitations of the LC‒MS/MS assay and warrants inclusion in future studies. Finally, sample sizes were unbalanced across groupings and time points, with uneven representation of women (~22%), which introduces sex-related bias.

Despite these limitations, this unique study provides valuable insights into distinct temporal differences across COVID-19 severity groups. We show that critically ill patients are characterized by early increases in microbial translocation and gut barrier damage biomarkers and pro-inflammatory cytokines, and that these factors remain elevated over approximately the first week of hospitalization compared to moderately and severely ill patients. This is coupled with early loss of commensal gut bacteria and followed by an expansion of many opportunistic pathogens. These early markers may prove valuable as predictors of critical illness. Further, attempting to address gut barrier damage and microbial dysbiosis early in critically ill patients’ hospitalization may help improve systemic inflammation and overall health outcomes. One strength of this study is the thorough profiling of fatty acids and bile acids. The progressive increase of branched-chain fatty acids in critical patients to our knowledge has not been shown to this detail, and we believe is reflective of a switch to a high-protein diet and amino acid fermentation by gut microbes and warrants additional investigation.

## Methods

### Study design

In this longitudinal study, we enrolled 22 COVID-19-infected patients between January and March 2021 at Sacco Hospital in Milan, Italy. Patients donated a combination of blood plasma, rectal swabs, and nasal swabs at up to 3 separate timepoints (referred to as T0, T1, and T2). Samples were collected between January and March 2021 (Supplementary Figure 1A), with an average of 3.5 d (SD = 1.0) between the baseline sample (T0) and the second sample (T1), and an average of 8.5 d (SD = 2.3) between the baseline sample and the final sample (T2). Timepoints were spaced to capture clinically meaningful changes over a short hospitalization window.

At the time of sample collection, COVID-19 illness was classified as moderate, severe and critical, according to World Health Organization (WHO) definitions.^62^ Moderate illness was defined as patients with modest supplemental oxygen requirements (nasal cannula), severe illness with respiratory deficiency requiring high-flow oxygen support (i.e., Venturi mask), and critical illness was defined as patients with respiratory failure requiring continuous positive airway pressure (C-PAP) or mechanical ventilation. The study protocol and data collection methods were approved by the Review Board of Ospedale Luigi Sacco Institution (n.prot 2020/ST/049), ensuring the protection of human subjects and adherence to ethical standards. All patients provided informed written consent, and all procedures were performed in compliance with relevant laws and institutional guidelines and were approved by the appropriate institutional committee(s).

### 16S rRNA gene amplicon sequencing

DNA and RNA were extracted from nasal and rectal swabs using the Qiagen AllPrep PowerViral DNA/RNA extraction kit according to the manufacturer’s instructions. Library preparation and sequencing were performed at the University of Minnesota Genomics Center as previously described.^17^ ^,^ ^63^ Briefly, the V3V4 locus of the rRNA gene was amplified in a 25-cycle PCR followed by a 10-cycle PCR to attach dual-indexed sequencing barcodes and adapters.^64^ Sequencing libraries were normalized, pooled, and loaded onto an Illumina MiSeq using a 2 × 300 v3 flow cell (Illumina, San Diego, CA) and sequenced to an average depth of 24,644 reads per sample.

Primers were removed from the demultiplexed sequences using cutadapt^65^ and then quality filtered, trimmed, denoised, and merged in R (version 4.2.1) using the dada2^66^ package. Merged sequences were further filtered to include only those within the expected base-pair length of the V3V4 amplicon and then used to generate an Amplicon Sequence Variant (ASV) table. Taxonomy was assigned using the SILVA v138 database.^67^ Unassigned ASVs or those belonging to Archaea, Eukaryota, Mitochondria, and Chloroplast were discarded. The decontam^68^ R package was then used to filter out any ASVs that were possible contaminants based on their prevalence in reagent-only negative control samples. ASVs were further filtered, requiring a prevalence of at least 10 reads in 10% of the samples, followed by removal of any samples with fewer than 500 total filtered reads. The filtered ASV tables for rectal and nasal swabs were used for all downstream analyses.

### SARS-CoV-2 viral loads

Quantification of N1 SARS-CoV-2 viral loads in nasal and rectal swabs was performed using the One-Step RT-ddPCR Advanced Kit for Probes (Bio-Rad 1864022). Briefly, 5 µL of sample was combined with 5 µL of One-Step RT ddPCR supermix, 1 µL of 300 mM DTT, 2 µL of N1 primers (obtained from IDT primer kit 10006713), 2 µL of Bio-Rad RT polymerase, and 5 µL of RNase/DNase free water. The cycling conditions were as follows: reverse transcription, 45 °C (60  min); enzyme activation, 95°C (10  min); 40 cycles, each, of denaturation, 95 °C (30 sec), and annealing, 60 °C (1 min); enzyme deactivation, 98 °C (10 min); and holding, 4 °C (infinite). All temperature changes were performed with a ramp speed of 2°C/s per the manufacturer’s recommendations.

### Cytokines and gut barrier markers

Plasma cytokine levels were assessed using two multiplex immunoassay platforms as previously described.^17^ IL-1β, IL-2, IL-6, IL-10, IL-12p70, IL-18, TNF-*α*, and IFN-*γ* were measured using the ProteinSimple SimplePlex system, while IL-8, IL-17A, and IL-23 were quantified using the MILLIPLEX High Sensitivity T Cell Panel. Biomarkers associated with microbial translocation and gut barrier damage were measured using commercially available ELISAs including LBP (Cell Sciences CKH113), sCD14 (R&D Systems QK383), zonulin (MyBioSource MBS706368), and I-FABP (R&D Systems DFBP20). Measurements below the assay detection limits were imputed using the lowest interpolated concentration within the dataset.

### Plasma metabolomics

For the determination of fatty acid concentrations, plasma samples were processed as previously described.^17^ ^,^ ^69^ Briefly, plasma samples were spiked with a mix of deuterated internal standards and subjected to protein precipitation using cold methanol. After centrifugation, the supernatant was derivatized with 3-nitrophenylhydrazine and 1-ethyl-3-(3-dimethylaminopropyl) carbodiimide, incubated at 40 °C, and diluted with water/acetonitrile prior to analysis. Targeted metabolite quantification was performed by LC–MS/MS in negative ion mode using multiple reaction monitoring on an Agilent 1290 Infinity II LC system coupled to an Agilent 6495 C mass spectrometer. Chromatographic separation was achieved on an Acquity UPLC BEH C18 column at 60 °C using mobile phases consisting of methanol/water (A; 1/1; v/v) and methanol/isopropanol (B; 1/1; v/v).

For quantification of bile acids in plasma, isotopically labeled bile acids (20  μL) were spiked into 100  μL of plasma. Bile acids were then extracted using 300  μL of 90:10:v/v cold methanol/acetonitrile and mixed at 1200 rpm for 15 min at 4 °C. The samples were subsequently centrifuged at 14,000xg for 20 min at 4 °C, and the supernatant was removed and dried under nitrogen gas. The samples were reconstituted in 100  μL of 30:70 v/v methanol/water and mixed thoroughly for 1 min. The samples were then centrifuged for 20 min at 14,000xg and the clear supernatant was transferred to a glass insert for LC‒MS/MS analysis using a Shimadzu LC-20AD XR Prominence LC system (Kyoto, Japan) with an Acquity C18 BEH (2.1 mm × 50 mm, 1.7 µm) column coupled to a Sciex 5500 QTRAP (Framingham, MA) mass spectrometer in negative ion-mode. Protocols were closely followed as previously performed regarding LC gradient and MS parameters.^70^ Briefly, the mobile phases consisted of A- 10 mM ammonium acetate in 900 mL water, 100 mL methanol, 1000 μL ammonium hydroxide (25%), B- 10 mM ammonium acetate in 1000 mL methanol, and 1000 μL ammonium hydroxide (25%). The following gradient was used: 80% A for 0.05 min, a stepwise linear decrease to 53% A at 0.1 min, to 49% A at 2.0 min, and to 28% A at 4.5 min. The flow rate was set to 0.5 mL/min. A column wash at 100% B for 0.5 min and re-equilibration at 100% A for 0.6 min were performed at a flow rate of 0.8 mL/min. The dwell times were set to 20 milliseconds, and both Q1 and Q3 were operated at unit resolution in MRM mode. The acquired data for all fatty acids and bile acids were analyzed using Agilent MassHunter and Skyline, respectively. The undetected values were replaced with the lowest interpolated value in the dataset.

### Microbiome analysis

Alpha diversity metrics (Shannon index and observed features) were calculated using the microbiome^71^ R package and analyzed as described below. Beta diversity of the rectal and nasal microbiomes was evaluated using the relative abundances of the filtered ASV tables to determine Bray‒Curtis distances, and differences between severity groups were tested at each time point by a PERMANOVA using the *adonis2* function in the vegan^72^ R package. Microbiome stability was calculated on the basis of within-patient Aitchison distances between timepoints and tested with pairwise Wilcoxon tests. Differentially abundant taxa at the genus and species levels were tested with the MaAsLin2^73^ R package using the relative abundances of the filtered ASV table. For the MaAsLin2 analysis, a composite variable of severity-timepoint and gender was included as a fixed effect and patient ID as a random effect, using moderate_T0 as the reference level.

### Statistical analysis

Since samples were collected after varying amounts of time during each patient’s hospitalization, the average amount of time between the first sample collected and subsequent samples was tested for differences between severity groups using a one-way ANOVA in GraphPad Prism (version 10.2.3). A Fisher’s exact test was used to compare the proportions of gender between severity groups using the stats package in base R. Concentrations of plasma cytokines, gut barrier integrity markers, and metabolites were log_10_-transformed prior to analysis, and ddPCR viral loads were log_10_-transformed with an added pseudocount of 1 due to the presence of zeroes. A linear mixed effects model using the lmerTest^74^ R package, followed by pairwise comparisons with the emmeans R package, was used to assess differences between COVID-19 severity groupings for all cytokines, gut barrier integrity markers, metabolites, and alpha diversity metrics. For each model, severity, time point, and gender were treated as fixed effects, and patient ID was treated as a random effect, using an interaction term between severity and time point. The reference levels for each of the fixed effects were moderate severity, baseline (T0) time point, and female gender. The assumptions of the final model (e.g., normality of the residuals and homogeneity of variance) were visually evaluated using the performance^75^ R package. The rmcorr^76^ R package was used to test for repeated measures correlations between rectal swab taxa abundances, swab viral loads, plasma cytokines, gut barrier integrity markers, and metabolites. Correlations were performed using the log-transformed biomarker concentrations and centered log-ratio (CLR) transformed taxa abundances. A correlation network was constructed in Cytoscape (version 3.10.0), to visualize correlations with a nominal *p*-value < 0.05 and a correlation coefficient greater than 0.4 or less than −0.4. Significance for all other tests was determined using a nominal *p*-value of 0.05.

## Supplementary Material

Supplemental_Figures.docx Supplemental_Figures.docx

Manuscript_Supplemental_Tables_resubmission.xlsx Manuscript_Supplemental_Tables_resubmission.xlsx

## Data Availability

Sequencing data for the 16S rRNA analysis are available at NCBI under BioProject ID PRJNA1289155. All code and raw data used for this analysis are available upon request.

## Abbreviations

US: United States
COVID-19: Coronavirus Disease 2019
SARS-CoV-2: Severe Acute Respiratory Syndrome Coronavirus 2
ACE2: Angiotensin-Converting Enzyme 2
SCFA: Short-Chain Fatty Acid
MCFA: Medium-Chain Fatty Acid
BCFA: Branched-Chain Fatty Acids
T0: Timepoint 0/Baseline
T1: Timepoint 1
T2: Timepoint 2
IRB: Institutional Review Board
IL: Interleukin
TNF: Tumor Necrosis Factor
IFN: Interferon
LBP: Lipopolysaccharide Binding Protein
sCD14: Soluble Cluster of Differentiation 14
I-FABP: Intestinal Fatty Acid Binding Protein
LC: Liquid Chromatography
MS: Mass Spectrometry
MRM: Multiple Reaction Monitoring
ddPCR: Droplet Digital Polymerase Chain Reaction
CLR: Centered Log-Ratio
PERMANOVA: Permutation Multivariate Analysis of Variance
ANOVA: Analysis of Variance
CA: Cholic Acid
CDCA: Chenodeoxycholic Acid
UDCA: Ursodeoxycholic Acid
DCA: Deoxycholic Acid
HDCA: Hyodeoxycholic Acid
GDCA: Glycodeoxycholic Acid
TCDCA: Taurochenodeoxycholic Acid
TDCA: Taurodeoxycholic Acid
GUDCA: Glycoursodeoxycholic Acid
GCDCA: Glycochenodeoxycholic Acid
GCA: Glycocholic Acid
TCA: Taurocholic Acid
GHDCA: Glycohyodeoxycholic Acid
ICU: Intensive Care Unit
FXR: farnesoid X receptor
TGR5: Takeda G protein-coupled receptor 5
