# Supplementary material for: Longitudinal multi-omic dynamics in hospitalized COVID-19 patients based on disease severity
Source: Gut Microbes Rep. 2026 Aug 13;3(1):2712728. doi: 10.1080/29933935.2026.2712728 (PMC13475324; doi:10.1080/29933935.2026.2712728)
Supplement: Supplementary file 1 [file KGMR_A_2712728_SM2857.docx]

**Supplemental Figure 1.** Samples collected for each patient enrolled in the study including nasal swabs, rectal swabs, and blood plasma (A). Bars represent the length of time between samplings, with symbols depicting when swab and blood samples were collected. All samples were collected from January-April 2021. Number of days between matching baseline samples and the first time point (B) and second time point (C) with each bar showing the mean and standard deviation for each group. Difference in gender composition between severity groups (D).

**Supplemental Figure 2.** Concentrations of sCD14 (A), I-FABP (B), zonulin (C), IL-8 (D), TNFα (E), IL-10 (F), IL-2 (G). Pairwise comparisons between groups at each time point were performed using the emmeans R package, nominal p-values < 0.05 were considered significant. Box plot whiskers extend to the min and max values with median plotted in center of the box.

**Supplemental Figure 3.** Comparisons of the total concentrations of short-chain fatty acids (SCFAs), branched-chain fatty acids (BCFAs), and medium-chain fatty acids (MCFAs) between severity groups overtime. Pairwise comparisons between groups at each time point were performed using the emmeans R package, nominal p-values < 0.05 were considered significant. Box plot whiskers extend to the min and max values with median plotted in center of the box.

**Supplemental Figure 4.** Nasal swab (A) and rectal swab (B) SARS-CoV-2 N1 gene copies/µL measured by ddPCR. Comparison of N1 gene copies/µL between swab types at each timepoint, and within swab types over time using mixed-effects analysis and uncorrected Fisher’s LSD test for pairwise comparisons (C). All N1 copies/µL were log_10_(x+1) transformed prior to analysis. A nominal p-value < 0.05 was considered significant.
